# Teratology – past, present and future

**DOI:** 10.2478/v10102-012-0027-0

**Published:** 2012-12

**Authors:** Eduard Ujházy, Mojmír Mach, Jana Navarová, Ingrid Brucknerová, Michal Dubovický

**Affiliations:** 1 Institute of Experimental Pharmacology & Toxicology, Slovak Academy of Sciences, Bratislava, Slovakia; 2 1^st^ Department of Pediatrics, Medical School Comenius University, Bratislava, Slovakia

**Keywords:** teratology, history, congenital developmental disorders, principles

## Abstract

Teratology is the science that studies the causes, mechanisms, and patterns of abnormal development. The authors present an updated overview of the most important milestones and stages of the development of modern teratology. Development of knowledge and society led to the recognition that causes of congenital developmental disorders (CDDs) might be caused by various mechanical effects, foetal diseases, and retarded or arrested development of the embryo and foetus. Based on the analysis of the historical development of hypotheses and theories representing a decisive contribution to this field, we present a survey of the six Wilson′s fundamental principles of teratology. The aim of observing these principles is to get insight into developmental relations and to understand mechanisms of action on the level of cell populations (elementary morphogenetic processes), tissues and organs. It is important to realise that any negative intervention into the normal course of these processes, either on genetic or non-genetic basis, inevitably leads to a sequence of subsequent changes resulting in CDDs. Moreover, the classical toxicologic monotonic dose-response paradigm recently has been challenged by the so-called “low dose-hypothesis”, particularly in the case of endocrine active substances. These include some pesticides, dioxins, polychlorobiphenyls (PCBs), and bisphenol A. Despite modern approaches of molecular biology and genetics, along with top diagnostic techniques, we are still not able to identify the actual cause in more than 65 to 70% of all congenital defects classified as having an unknown etiology. Today CDDs include any birth defect, either morphological, biochemical, or behavioural.

## Introduction

Teratology started as a descriptive science, stemming from a variety of mystical and scientific theories explaining the etiology of congenital malformations, such as maternal impression, the position of the stars, hybridisation, etc. While superstitions and fantastic explanations of congenital developmental disorders (CDDs) prevailed, there existed also biological theories which seem to be rational today. Further development of knowledge and society led to the recognition that causes of CDDs were manifold. Various mechanical effects, foetal distress, retarded or arrested development of embryo and foetus and several chemical substances and physical factors may come into play. In the beginning of our short review we present an overview of historical development, key persons and milestones in biological theories of CDDs:

- **W. Harvey** (1578–1657) - used the term “developmental arrest”,
- **C.F. Wolff** (1733–1794) - in his study on the intestine, the term “germ layer” was coined that has been in use to this day,
- **A. von Haller** (1708–1777) - was first to describe the development of the chicken heart,
- **I.G. de Saint-Hillaire** (1805–1861) - was first to introduce the term “teratology”,
- **C. Dareste** (1822–1899) - discussed the modes of artificial induction of monstrosities (particularly by mechanical impulses during icubation of hen eggs),
- **R. Virchow** (1821–1902) - gathered a unique collection of rare developmental disorders of the human body in the “Museum of Pathology” in the Berliner hospital Charité,
- **E. Schwalbe** (1906–1999) - defined the expression “teratogenic termination point”,
- **CH.R. Stockard** (1879–1936) - introduced the term “critical period”.

Teratology as a modern science was born in the 1930s with the publication of a set of experiments in which pregnant pigs were fed a diet deficient in vitamin A. All of these piglets suffered a variety of malformations, predominantly a lack of eyes (Hale, 1933; 1935).

He has concluded that a nutritional deficiency leads to a marked disturbance of the internal factors which control the mechanism of eye development. The physician Josef Warkany is considered the father of experimental teratology. In the 30s and 40s of the past century, he was the first to prove that CDDs can be induced by exogenous factors also in mammals. His studies led to the definition of both genetic and environmentally induced structural defects (Warkany & Nelson, 1940; Warkany & Schraffenberger, 1947). The susceptibility of mammalian embryos to toxicity from xenobiotic agents was demonstrated in a series of studies in experimental animals with congeners of biologically active molecules, such as amino acid mimicking azaserine (Tiersch, 1957; Friedman, 1957). A human counterpart to these experiments was reported in the 1950s, when aminopterin was used in human pregnancy to produce abortion (Thiersch, 1952). In further experiments, various physical factors were used: radiation (Brent, 1960; Rugh, 1963), changes in temperature (Pennycuik, 1965; Skreb, 1965), hypoxia (Ingalls *et al.,* 1950; Murakami & Kameyama, 1963), hormones – estrogens (Green *et al.,* 1939; 1940), androgens (Biggs & Rose, 1947; Grunback & Ducharme, 1960), cortisone (Fraser & Fainstat, 1951; Gunberg, 1958), hypovitaminosis – riboflavin (Warkany & Nelson, 1940; Aksu *et al.,* 1968), folic acid (Nelson & Evans, 1949; Nelson *et al.,* 1952), hypervitaminosis – vitamin A (Cohlan, 1953; Giroud & Martinet, 1954), vitamin D (Warkany, 1943; Friedman & Roberts, 1966), but also drugs and a number of other chemical substances. In this period, publications appeared reporting on the effect of the environment and genetics, as well as their mutual combinations, as the causes of malformations in experimental animals (Wilson, 1959; Giroud & Tuchmann-Duplessis, 1962). The major events that contributed to our knowledge in teratology prior to the thalidomide catastrophe are listed in Table 1.

**Table 1:** Historical events in modern teratology.

Year	Historical event
1905	The first experimentally induced developmental toxicity in mammals. Embryonic lethality induced by X-rays in cats (Tousey).
1921	The first experimentally induced teratogenicity in mammals. Disorders in limbs in pigs induced by lipid diet (Zilva *et al.*).
1929	The first description of malformations in humans caused by exogenous factors. Microcephalia caused by X-ray irradiation of the pelvis (Goldstein and Murphy).
1935	Recognition of food deficiency leading to malformations in animals. Eye disorders in pigs due to hypovitaminosis A (Hale).
1937	Hormones causing alterations in sexual differentiation in animals. Masculinisation of female foetuses in mice due to the action of androgens (Raynaud).
1941	Report on virus-induced human malformations. Rose-rash induced eye disorders (Gregg).
1944	The first evidence of postnatal effect following prenatal administration of a chemical substance. Decreased learning ability in rats caused by the administration of sodium bromide (Hamilton and Harned).
1948	General recognition of chemically induced teratogenicity. Experiments with alkylating agents (Haskin) and trypan blue (Gillman *et al.*).
1952	The first report on malformations caused by drugs in humans. Multiple malformations in foetuses caused by aminopterin (Thiersch).
1959	The first report on human malformations induced by environmental pollutants. Disorders of the central nervous system and dentition caused by methyl mercury (Kitamura *et al.*).
1961	Thalidomide-induced embryopathy

Adapted according to Schardein (1988) Teratological testing: status and issues after two decades of evaluation. Rev Environ Contam Toxicol pp. 1–78.

The **thalidomide episode** of the early 1960s increased our understanding of developmental toxicology, providing a clear example of an agent that produced minimal toxicity to adults but a high degree of embryotoxicity. Maternal exposure to the mild sedative-hypnotic agent thalidomide (useful for nausea and vomiting and effective against influenza) was suspected to be causing characteristic reduction deformities of the limbs, ranging from hypoplasia of one or more digits to the total absence of all limbs. An example of the thalidomide embryopathy is phocomelia (the structures of the hand and feet may be reduced to a single small digit, or may appear virtually normal but protrude directly from the trunk, like the flippers of a seal-phoca). The proof was presented by the independent discoveries of Lenz (1961) and McBride (1961), which led to the worldwide interest in clinical teratology. In September 1961**,** the German scientist, Wiedemann published a scientific paper delineating the clinical syndrome, calling attention to the increase in the incidence of hypoplastic and aplastic malformations (phocomelia) of the extremities. This was the first publication alarming the scientific world to the defect.

The aim of experimental teratology in the **post-thalidomide period** has been the exact explanation of causes and mechanisms of the rise of CDDs. Wilson (1973) defined teratology as a science dealing with adverse effects of the environment on developing systems, namely on germ cells, embryos, foetuses, and immature individuals. A more comprehensive definition is that teratology is the science dealing with the causes, mechamisms, and manifestation of developmental deviations of either structural or functional nature. He formulated a concept of six main principles of teratology that are generally accepted to this day.

1. Susceptibility to teratogenesis depends on the genotype of the conceptus and the manner in which this interacts with adverse environmental factors.
2. Susceptibility to teratogenesis varies with the developmental stage at the time of exposure to an adverse influence.
3. Teratogenic agents act in specific ways (mechanisms) on developing cells and tissues to initiate sequences of abnormal developmental events (pathogenesis).
4. The access of adverse influences to developing tissues depends on the nature of the influence (agent).
5. The four manifestations of deviant development are death, malformation, growth retardation, and impaired function.
6. Manifestations of deviant development increase in frequency and degree as dosage increases from no-effect to the totally lethal level.

In the following period of teratological research, major emphases have been placed on the causes and mechanisms of abnormal development since recognition and understanding of these aspects are likely to be most helpful in taking preventive measures. Quantitative and distributional aspects of epidemiology have been included because, by throwing light on causes and mechanisms, they also contribute to the prevention of CDDs (Wilson & Fraser, 1977; Kalter, 2003).

Prof. Richard Jelínek, a Czech teratologist, came with the “revised” principles of teratogenesis (Jelínek, 2005). His concept was based upon the idea that the basic unit of individual development and teratogenesis is not the single cell but the morphogenetic system defined as a set of cell populations carrying, creating and performing the programme for the development of definitive body parts. Within local cell populations, four basic morphogenetic processes are operative (Rychter & Jelínek, 1978). These sequences characterise the development of any embryonic component:

- Cellular proliferation
- Distribution
- Integration into higher entities by means of cell contacts
- Reduction of cell numbers along the pathways of selective cell death.

At present, it is known that all responses of the cell are mediated through its genome. Mendelian heredity is common in cases of congenital metabolic disorders that are based on a mutation in the sequence encoding a certain enzyme. Molecular mechanisms have also been implicated in some of the known teratogens, such as thalidomide, retinoids, valproic acid, and cancer chemotherapeutic agents (Schardein, 2000; Finell *et al.*, 2002). Since little is known as yet about the basic process regulating development, the exact mode of action of reproductive toxicants, embryo/foetotoxic agents or teratogenic compounds is seldom known. Reproductive toxicants may cause one or more of the following types of changes (Wilson, 1973; Niesing *et al.,* 1996):

- mutations
- chromosomal aberrations
- disturbances in cell division
- changes in nucleic acid composition and protein synthesis
- reduction in the amount of essential constituents for biosynthesis
- reduction of energy supply for embryonic and foetal development
- disturbances of enzyme systems
- disturbances in the regulation of water and electrolyte balance
- changes in membrane characteristics

The causes of birth defects are varied, yet the aetiology of most malformations is unknown (Table 2, Brent & Beckman, 1990).

**Table 2:** Suspected causes of birth defects in humans.

Suspected cause	% total
**Genetic**	
Autosomal genetic disease	15–20
Cytogenetic	5
**Environmental**	
Maternal conditions	4
Maternal infections	3
Mechanical problems (deformations)	1–2
Chemicals/drugs/radiation/hyperthermia	<1
**Preconception exposures**	?
**Unknown (polygenic**)	65

In 1990, two networks of teratology information services were established, in Europe ENTIS (European Network of Teratology Information Services, www.entisorg.com) and in the USA OTIS (Organization of Teratology Information Specialists, www.otispregnancy.org). A teratology information service provides health professionals and patients with “tailor-made” information relating to the pertinent situation, illness and chemical exposure of the individual involved (Schaefer *et al.,* 2005).

## *In vitro* methods

Current applications of *in vitro* developmental systems can be broadly divided into two areas: (1) prescreening for developmental toxicants and (2) testing for elucidation of mechanisms of normal and abnormal embryogenesis (Hood, 2006). At the 17^th^ meeting of the European Centre for the Validation of Alternative Methods (ECVAM) Scientific Advisory Committee in 2001, three methods were endorsed as “scientifically validated” and ready for consideration for regulatory acceptance and application, and therefore deserve a more detailed discussion.

These are:

- **Embryonic Stem-Cell Test (EST)** – two permanent murine cell lines are used - D3 cells represent embryonic tissue and 3T3 fibroblast cells represent adult tissue (Spielman *et al.,* 1997; Genschow *et al.,* 2000).
- **Micromass Test (MM)** – is based upon rat embryonic limb mesenchyme cells, when cultured in small volumes at high density, from foci of differentiating chondrocytes within a background of non-differentiating cells (Flint & Orton, 1983).
- **Whole Embryo Culture Test (WEC)** – this teratogen screening system is using whole mouse **(**Sadler *et al.*, 1982), rat (Schmid, 1985) and rabbit embryo (Ninomiya *et al.*, 1993). Embryos cultured for short periods during the phase from fertilisation to the end of organogenesis.

## Functional and neurobehavioural teratology

The first experimental proof that a chemical substance can adversely affect neurobehaviuoral development was reported by Hamilton & Harned (1944). They found that sodium bromide administered prenatally caused decreased ability of spatial learning in adult rats. Werboff & Gottlieb (1963) were the first to present the idea that chemical substances acting during prenatal development can affect the behaviour of an individual during the postnatal period, and they formulated the fundamentals of a new teratological discipline, the so-called behavioural teratology. During the 70s and 80s of the 20th century, several chemical substances were identified along with other factors acting as functional and/or behavioural teratogens, e.g. alcohol, certain addictive substances, heavy metals, X-ray radiation, and environmental pollutants (Grandjean & Landrigan 2006). Recently, the terms neurobehavioural teratology or developmental neurotoxicology have been preferred. These terms, however, represent a more complex study of causes and mechanisms (the so-called toxicity pathways) of damage to the developing brain (Bushnell *et al.* 2010).

Further development of neurobehavioural teratology is considered a very important issue. It is associated with the so far unexplained increasing rate of psychic and behavioural disorders on the one hand, and with excessive chemisation and the action of excessive stressful stimuli on the other. According to several experts, the extremely increasing number of individuals with an autistic spectrum of disorders recorded in the U.S.A. over the past 20 years cannot be explained only by improved diagnostics and changed diagnostic criteria (Kim *et al.* 2011). It is assumed that a number of chemical substances and their mutual interactions, with no negative effect on the adult brain, may over their long-term action negatively affect the developing brain even at very low concentrations.

According to Prof. Günter Dörner (2004), the nervous, endocrine, immune and reproductive systems form a mutually functionally interconnected neuroendocrine-immune system. During critical developmental stages, various hormones, neurotransmitters and cytokines play a key role in the functional development of the respective physiological systems as so-called ontogens or developmental signals and organizers. Various environmental factors may, directly or indirectly, affect the respective developmental processes, such as cell proliferation and migration, development of neurites, myelinisation, synaptogenesis, or apoptosis. They can however also act together with the activity of biologically active signal substances, which may subsequently lead to nonphysiological concentrations. These changed concentrations of ontogens may act as so-called endogenous or functional teratogens (Dörner, 2004). Functional changes in the brain may become manifested as various behavioural, emotional, or cognitive disorders. Some of the functional disorders/changes need not manifest under basic conditions but appear after the action of certain physiological burdens, such as alcohol and drug abuse, polluted environment, or the action of excessive stress (Dubovicky, 2010).

Since the 90s of the 20th century, there has been growing concern of a permanent damage to the endocrine and nervous system after developmental exposure to endocrine disrupting chemicals, which led to the study of low-dose effects and non-monotonic-dose response phenomena. Exposure to endocrine disruptors during early life may cause long-term health effects and can influence both the reproductive and neurobehavioural development of the offspring, even until maturity or middle age (Patisaul & Adewale, 2009; Belloni *et al.,* 2011). Persistent developmental toxicity after low-dose exposure to a mixture of endocrine disrupting pesticides was found in young and adult male offspring. The toxicity becomes manifested by reduced prostate, epididymis and testis weights altered prostate histopathology, reduced sperm counts and decreased spatial learning. As no significant effects were seen following single-compound exposure, these results indicate adverse effects of mixtures at dose levels where the single pesticide did not exert significant effects (Jacobsen *et al.,* 2012). Over-chemisation of the environment becomes an important challenge for further research and development of environmental and so-called “mixture toxicology” (Mumtaz, 2010).

There are various definitions of the low-dose effect toxicity. Generally, it is any dose below the lowest observed effect level or the lowest observed adverse effects level (NOAEL). Low-dose toxicity closely relates to non-monotonic dose response with specific inverted U-shaped, U-shaped, and multiphasic curves. Experimental as well as epidemiological studies showed several chemical compounds with low-dose toxicity and non-monotonicity profile, such as bisphenol A, atrazine, dioxines and perchlorates (Richter *et al.,* 2007; Hayes *et al.,* 2011; Mocarelli *et al.,* 2011; Zoeller, 2010). A large number of studies have focused on the effects of bisphenol A on the brain and behaviour with the most significant effects on sexually dimorphic regions of the brain and behaviours. Other affected behaviours included social behaviour, learning and anxiety and maternal-neonate interactions (Vandenberg *et al.,* 2009). Low-dose toxicity and non-monotonicity have become highly topical in functional and neurobehavioural teratology.

Development of new technologies has brought also new chemical entities with a potential to affect biological systems. Nanotechnology and bioelectronics are rapidly growing branches of industry. Nanoparticles and gradually decomposed electronic particles inserted to the body may represent risks for the living system, especially during development (Blum *et al.,* 2012). Research of new technologies will urge the need for testing new chemicals for possible functional and neurobehavioural teratogenicity. Recent experimental studies have shown that nanoparticles can interfere with developmental processes and may affect reproductive and other important physiological functions (Amorim & Scott-Fordsmand, 2012). Reproductive and developmental risk assessment will thus be highly topical in the near future.
